# Identification of Differentially Expressed Genes Related to the Lipid Metabolism of Esophageal Squamous Cell Carcinoma by Integrated Bioinformatics Analysis

**DOI:** 10.3390/curroncol30010001

**Published:** 2022-12-20

**Authors:** Meng-Ying Cui, Xing Yi, Dan-Xia Zhu, Jun Wu

**Affiliations:** Department of Oncology, The Third Affiliated Hospital of Soochow University, 185 Juqian St, Changzhou 213003, China

**Keywords:** ACSL1, OIP5, lipid metabolism, ESCC

## Abstract

Purpose: In recent years, lipid metabolism has been reprogrammed to meet the energy and substrate needs of tumorigenesis and development and is a potential new target for cancer treatment. However, the regulatory mechanism of lipid metabolism in esophageal squamous cell carcinoma is not well understood. Methods: We first downloaded the esophageal squamous cell carcinoma (ESCC) gene dataset in the GEO and TCGA databases and analyzed the central differentially expressed genes (DEGs) of ESCC through bioinformatics. Afterwards, the GSEA method was used to analyze the lipid metabolism-related pathway of the central gene in the pathological process of ESCC, and it was determined that the central gene OIP5 was significantly related to the fatty acid metabolism pathway. Our heatmap also revealed that the enrichment of the ACSL family in ESCC tissues was more pronounced than in normal tissues. We hypothesized that OIP5 can regulate the fatty acid metabolism process in ESCC cells and affect the tumorigenic ability of ESCC. Further statistical analysis and experiment were conducted to determine the lipid metabolism-related gene, OIP5′s, expression pattern and clinical significance in ESCC, analyze the effect of OIP5 expression on fatty acid metabolism-related enzymes in ESCC, revealing the specific mechanism of OIP5 that promotes ESCC development. Conclusions: Our study established a correlation between OIP5 expression and clinicopathological factors (tumor size, T stage, N stage, and clinical grade) in esophageal squamous cell carcinoma (*p* < 0.05). We have also experimentally demonstrated that OIP5 regulates ESCC fatty acid metabolism by influencing the expression of the key enzyme ACSL1 in lipid metabolism.

## 1. Introduction

Available studies have confirmed that cancer cells exhibit multiple genetic mutations at the somatic level, the nature of which is related to the genetic susceptibility of the individual organisms. Since then, many susceptibility genes and their polymorphisms have been identified for esophageal cancer, and polymorphisms of susceptibility genes have been identified, most of which are concentrated in gene regions related to carbohydrate and lipid metabolism and vitamin synthesis [1,2]. In patients with esophageal cancer, the rapid proliferation of tumor cells compared to normal cells and the increased energy requirements result in significant differences between the metabolism of tumor and normal tissues [3]. Previous studies have revealed that genes involved in lipid metabolism are highly expressed in malignant tumors and are considered as oncogenes [4,5]. Epidemiological studies have shown that obese individuals are at increased risk of malignancies, including esophageal squamous cell carcinoma, suggesting that fatty acid metabolism plays a key role in tumorigenesis and progression [5,6,7]. Fatty acids are one of the main energy sources in mammals and are crucial for the normal growth and metabolism of cells. However, the relationship between fatty acid metabolism and the pathological development of esophageal cancer remains unclear, and the relationship between lipid metabolism and the potential clinical prognosis of esophageal cancer has not been explored.

Opa-interacting protein 5 (OIP5), an Opa-interacting protein associated with eisseria gonorrhoeae opacity [8], is highly expressed in the human testis [9]. The upregulation of OIP5 is associated with adverse clinical features in multiple cancer types, including gastric cancer [10,11]. Downregulation of OIP5 has been reported to inhibit the proliferation of cultured colorectal and gastric cells [10]. Given the limited evidence for the association of OIP5 with oncogenic events, the mechanisms of OIP5-promoted tumorigenesis in esophageal cancer needs to be further investigated.

Our study demonstrated for the first time that OIP5 promotes the occurrence and development of esophageal cancer cells by mediating intracellular lipid metabolism. The study of the target gene OIP5 provides reference for the subsequent research of its function, mechanism of action, and clinical significance. However, the effect of OIP5 on fatty acid metabolism in esophageal squamous cell carcinoma has not been investigated. This study provides a new perspective on the role of intracellular fatty acid metabolism in the development of esophageal squamous cell carcinoma and may provide new insights into the diagnosis and potential treatment of esophageal cancer. It also presents a theoretical basis for the improvement of ESCC prognostic models, the elucidation of drug resistance mechanisms, and the development of corresponding targeted therapies.

## 2. Materials and Methods

### 2.1. Download ESCC-Related Expression Profile Dataset

Data from public databases were used in this study to analyze gene expression in patients with esophageal squamous cell carcinoma and healthy/control patients. GSE20347 and GSE75241 datasets were downloaded from the GEO database (https://www.ncbi.nlm.nih.gov/geo/ (accessed on 1 July 2020)). The GSE20347 dataset is based on the GPL571 platform ( [HG-U133A_2] Affymetrix Human Genome U133A 2.0 Array), while the GSE75241 dataset is based on the GPL5175 platform ([HuEx-1_0-st] Affymetrix Human Exon 1.0 ST Array [transcript (gene) version]). The array data for GSE20347 include 17 esophageal squamous cells and their matched normal tissues as control. The GSE75241 dataset include 15 esophageal squamous cell carcinoma samples and 15 non-malignant mucosal samples. In addition to these, in order to obtain a sufficient number of ESCC samples, we also obtained 80 ESCC samples and 79 normal control tissues to be compared against them from The Cancer Genome Atlas (TCGA) using the USCS Refseq Gene Array (https://www.gtexportal.org/ (accessed on 2 July 2020)) and 1 adjacent non-tumor tissue sample (https://tcga-data.nci.nih.gov/tcga/ (accessed on 2 July 2020)).

### 2.2. Differential Expression Genes Recognition

We used the downloaded data for background correction, using R to convert gene probe IDs from microarray data into genomic symbols in platform files. The dataset was normalized accordingly using the *t*-test ((Version 3.3, http://www.bioconductor.org (accessed on 14 October 2022))) of the linear model of the LIMMA package. We set the intercept value to |Log 2 FC| ≥ 1 (adjusted *p*-value < 0.05) to select differentially expressed genes between ESCC samples and normal control tissues. Next, we plotted the overlapping parts of the three datasets and found 307 differentially expressed genes (DEGs) by using a Venn plot (http://bioinformatics.psb.ugent.be/webtools/Venn/ (accessed on 26 August 2022)). At the same time, we performed gene ontology (GO) enrichment analysis on these differentially expressed genes to elucidate the GO functions of DEGs, such as biological processes (BP), molecular function (MF), and cellular components (CC). We also selected the KEGG enrichment pathway related to lipid metabolism to determine whether these DEGs were related to lipid metabolism in the development of esophageal cancer. A *p* < 0.05 was used as the cut-off standard for significant functions and pathways. We also performed a GSEA analysis to determine whether these differential genes are enriched in lipid metabolism in esophageal squamous cell carcinoma. Finally, we present a protein–protein interaction (PPI) network encoded by lipid metabolism-related genes OIP5 using the STRING online database (Version 10.0, http://string-db.org (accessed on 26 August 2022)) and the Cytoscape software (Version 3.8.0, http://www.cytoscape.org/ (accessed on 26 August 2022)).

### 2.3. Cell Culture, Cell Transfection and Construction of Gene Downregulation, and Overexpression Lentiviral Vectors

ESC-410 and KYSE-150 ESCC cell lines were purchased from the Shanghai Chinese Academy of Sciences Type Culture Collection Cell Bank. The two cell lines were cultured in culture flasks in growth medium RPMI1640 and incubated in an incubator (5% CO_2_, 37 °C) with 10% fetal bovine serum (Invitrogen, Waltham, MA, USA) + 1% penicillin streptomycin (Gibco, Gaithersburg, MD, USA). All experiments were repeated at least 3 times with both cell lines.

Plasmid-transfected viruses carrying the empty vector (pLVX-IRES-puro) and OIP5 and acyl-CoA synthetase long-chain family member 1 (ACSL1) gene vectors (three plasmid systems including pSAX2, pMD2G, and pLVX-IRES-puro-OIP5/ACSL1) were transfected using the helper dye Lipo3000 (Invitrogen, USA). The transfection was divided into 5 groups including the negative control group (Negative control, NC), the OIP5 and ACSL1 gene-overexpression groups (shRNA + RNA), and the OIP5 and ACSL1 gene-silencing groups (shRNA). Different lentiviruses (pLVX-IRES-puro-OIP5/ACSL1) and the same volume of control viral vector were added to them. Stable OIP5 and ACSL1 gene-silencing cell lines were obtained by screening ESC-410 and KYSE-150 ESCC cells with puromycin (Gibco, Gaithersburg, MD, USA) (2 ug/mL).

### 2.4. Tissue Microarray and Immunohistochemistry

To investigate the expression pattern of OIP5 in gastric cancer tissues, we used immunohistochemistry to detect tissue chips (chips were purchased from Shanghai Xinchao Biotechnology Co., Ltd. (Shanghai, China), including 2 normal esophageal mucosal tissues, 29 ESCC tissues, and the expression of OIP5 protein in paired 29 paracancerous tissues). It was found that OIP5 protein expression was significantly higher in ESCC tissues than in paraneoplastic tissues as well as in normal esophageal mucosal tissues. The results showed that there is significant difference in OIP5 expression between ESCC tissues and adjacent tissues.

### 2.5. RNA Extraction, cDNA (Complementary DNA) Synthesis and Real-Time PCR Analysis

Nude-mouse tumor tissue (50–100 mg) was ground into powder, added to 1 mL TRIzol lysis solution, and allowed to stand for 5 min at room temperature. After dissolving, it was transferred to a sterile centrifuge tube, and the supernatant was collected by centrifugation. The dead cells were washed in pre-chilled PBS and placed on ice after the cell culture media aspirated from the plate. We added 300–400 µL of TRIzol reagent to each well, mixed thoroughly until it became a homogeneous and translucent liquid state, and incubated it on ice for 5 min. The cells containing lysates in each well were pipetted into the EP tube, and the experimental group and the experimental group number were marked. We added 1/5 Trizol volume of chloroform, shook vigorously, and let it stand at room temperature for 3 min. The centrifuge was frozen at 4 °C, and centrifuged at 12,000 rpm for 30 min. The supernatant was transferred to a new EP tube, 50% triazole volume of isopropanol was added, gently mixed, and left at room temperature for 10 min. The centrifuge was set to 4 °C, at 12,000 rpm × 30 min, and the supernatant was discarded and then continued at 12,000 rpm × 5 min. A white RNA precipitate was observed in the EP tube. The same amount of 75% DEPC H_2_O ethanol was then added to the EP tube for washing. The centrifuge was set to 4 °C, at 7500 rpm × 5 min, and the supernatant was discarded, and the process was repeated twice. Then, the particles were dried, and a proper amount of DEPC H_2_O was added and incubated at 55–60 °C for 10 min to dissolve the precipitate and to obtain an RNA solution. RNA concentration was measured by NanoDrop spectrophotometer (Eppendorf, Hamburg, Germany).

Then, we used reverse transcription kits, which reverse transcribe RNA samples to cDNA.(Tiangen Biochemical Technology Co., Ltd., Beijing, China).

Finally, a real-time quantitative polymerase chain reaction analysis was performed using a real-time quantitative PCR kit (Tiangen Biochemical Technology Co., Ltd., Beijing, China). To perform RT-qPCR, 1 µL of cDNA was mixed with 1 µL of gene-specific forward primers, 1 µL of gene-specific reverse primers, and 10 µL of SYBR Green Real-time PCR Master Mix, and reactions were detected using the LightCycler 480 II instrument (Roche, Basel, Switzerland). The detailed RT-PCR reaction is shown below: 1 cycle of pre-denaturation at 95 °C for 30 min, followed by 40 cycles of denaturation at 95 °C for 15 s, annealing at 60 °C for 30 s, and extension at 72 °C for 30 s. Finally, the temperature was slowly raised to the range of 55–95 °C, and at each temperature, the sample was cycled for 30 s, for a total of 40 cycles. The CT values and related parameters of each group were recorded, and the relative gene expression levels were calculated by the comparative CT method (ΔΔ-CT). The primer sequences are shown in Table 1.

### 2.6. Quantification of Associated Lipids in ESCC

Different experimental groups were designed, and each group of cells was inoculated in 6-well plates at 5 × 10^5^ per well and placed in an incubator (37 °C, 5% CO_2_) for 24 h to reach 40–50% confluence. Cells were lysed in 5% Triton X-100 Cell Lysis Buffer. Intracellular neutral lipids were detected using BODIPY 493/503 dye (Thermo Fisher, Waltham, MA, USA) according to the manufacturer’s instructions. Intracellular triglyceride (TAG) and phospholipid (PL) content was measured using a triglyceride quantitative detection kit (BioAssay Systems, Hayward, CA, USA) and a phospholipid quantitative detection kit (BioAssay Systems), respectively.

### 2.7. Cell Proliferation Assay (CCK-8 Method)

To determine whether OIP5 affects the colony-forming ability of ESCC cells, we designed three groups including control groups (sh-NC), a silenced-OIP5 gene group (sh-OIP5), and an exogenously overexpressing-OIP5 gene group after silencing the OIP5 gene (sh-OIP5 + OIP5). Stably transfected ESCC cells were removed, digested, centrifuged, and resuspended, and 100 µL of cell suspension was inoculated in 96-well culture plates at a density of 2 × 10^3^ cells/mL. The plates were pre-incubated in an incubator (37 °C, 5% CO_2_) for 24 h and detected at 0, 24, 48, and 72 h. In addition, at the corresponding incubation time points, we added CCK8 solution (10 µL) to each well and placed the plate in an incubator (37 °C, 5% CO_2_) for 1 h. Finally, OD values (450 nm) were detected by a microplate reader, and cell growth curves were plotted in combination with the assay results at each time point.

### 2.8. Clone Formation Assay

Logarithmic growth phase cells were routinely digested, pipetted into individual cells and resuspended in DMEM medium (Gibco, USA) containing 10% fetal bovine serum. We diluted the cell suspension to 100 cells per dish. The density was inoculated in dishes containing 10 mL of pre-warmed medium, and the dishes were placed in an incubator (37 °C, 5% CO_2_) for 2 weeks. When macroscopic clones appeared in the petri dish, the culture process was considered to be terminated. The supernatant was discarded, and PBS was used for washing twice. We fixed with 4% paraformaldehyde for 15 min, discarded the fixative, and stained the colonies with Giemsa solution (Sinopharm Chemical Reagent, Shanghai, China) for 15 min, washed the staining solution with flowing water, and dried at room temperature. Colonies with more than 50 cells were counted under a microscope and histograms were drawn.

### 2.9. Mouse Xenograft

ESC-410 ESCC cells were stably transfected with OIP5 overexpression plasmid or empty vector, and untransfected ESC-410 cells were set as the control group. After PBS digestion and centrifugation of ESC-410 cells, cells were washed twice with PBS to remove serum. Cell pellets were pipetted to the appropriate concentration using serum-free medium.

**Figure 1:**
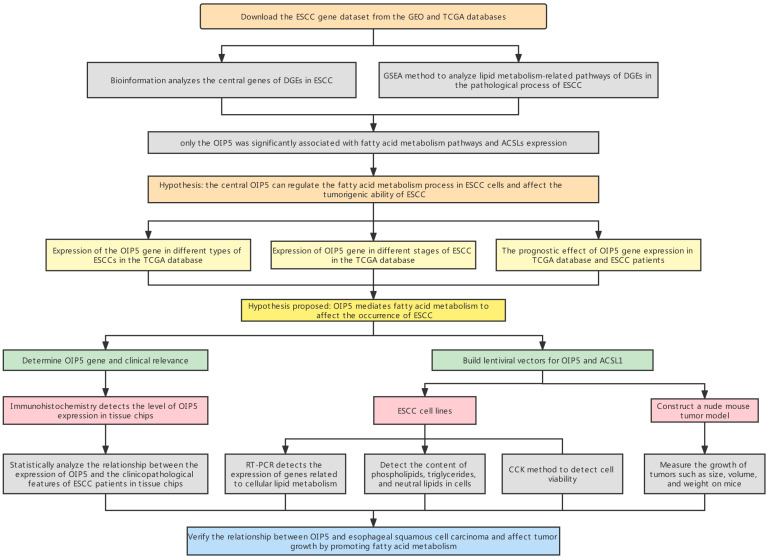
The study design.

Cells from different groups (approximately 2 × 10^7^ cells) were inoculated subcutaneously onto the back of BALB/c nude mice (5 weeks old, male). The date of inoculation was recorded as D0, and the tumor volume was measured on D5, D10, D15, D20, D25, and D30. The tumor volume was calculated using the equation: tumor volume (mm^3^) = 0.5 × (a × b^2^), where a corresponds to the tumor’s long diameter and b corresponds to the tumor’s short diameter. Tumor size was measured, and growth curves were plotted. After 30 days, nude mice were executed, subcutaneous tumors on the back of nude mice were excised for photographing and recording, and curves were plotted using tumor volume data recorded on D0, D5, D10, D15, D20, D25, and D30. Tumor size, weight, and gene expression patterns were further analyzed. All procedures were approved by Ethics Committee of Changzhou First People’s Hospital (The specific flow chart is shown in Figure 1).

### 2.10. Statistical Analysis

Data were statistically analyzed with Graphpad Prism 9.0 and expressed as mean ± standard deviation of at least three independent experiments. The data were analyzed using R software.

Quantitative data were subjected to a one-way ANOVA or two-sided *t*-test under normal distribution and chi-square, otherwise, the Wilcoxon rank-sum test was performed. A value of *p* < 0.05 was considered as statistically significant.

## 3. Results

### 3.1. Selection of DEGs in ESCC

**Figure 2:**
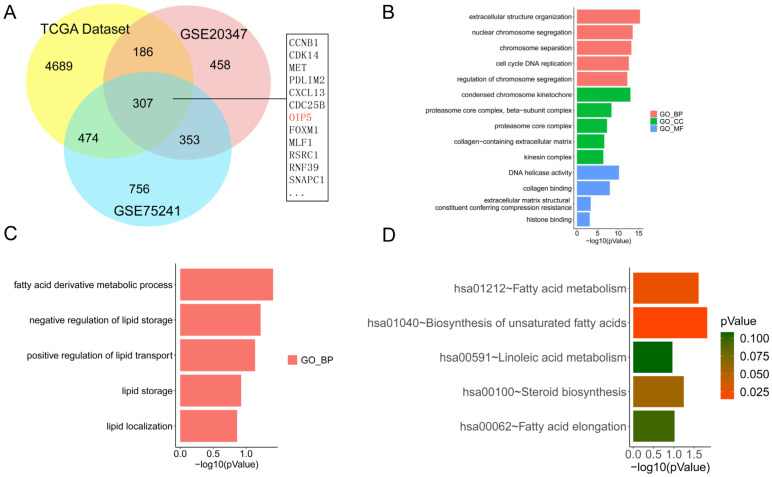
Venn chart of the DEGs in the GSE20347, GSE75241, and TCGA datasets (**A**). DEGs related GO and KEGG pathway enrichment (**B**–**D**).

To study the expression of OIP5 in ESCC, we downloaded the expression profiles of GSE20347 and GSE75241 from the GEO database. We obtained 1305 DEGs from the GSE20347 dataset, 1890 DEGs from the GSE75241 dataset, and 5656 DEGs from the TCGA database. Using Venny graph, we identified 307 genes in the overlap of three datasets, such as CCNB1, CDK14, MET, POLM2, CXCL13, CDC25B, OIP5, and FOXM (Figure 2A).

### 3.2. Enrichment Analysis of the Lipid Metabolism Pathway in ESCC

KEGG and GO functional enrichment analysis was used for DEGs functional analysis. The study found that 307 differentially expressed genes were significantly associated with the biological processes, cellular components, and molecular functions of ESCC. These genes are closely related with extracellular structure organization (GO:0043062), nuclear chromosome segregation (GO:0098813), chromosome separation (GO:0051304), and DNA replication (GO:0044786) (Figure 2B). The result suggests that these 307 DEGs are involved in various aspects of the occurrence and development of esophageal squamous cell carcinoma. In addition, we also selected lipid metabolism-related pathways, in which we found that these differentially expressed genes are also presented in the fatty acid-derivative metabolic process (GO:1901568), negative regulation of lipid storage (GO:0010888), positive regulation of lipid transport (GO:0032370), lipid storage (GO:0019915), and lipid localization (GO:0010876) (Figure 2C). The results of KEGG pathway analysis showed that these genes were enriched and upregulated compared to normal tissues in fatty acid metabolism, including, mainly, the degradation of fatty acids, the synthesis and elongation of unsaturated fatty acids, linoleic-acid metabolism, and the lipid-metabolism process in esophageal cancer (Figure 2D).

### 3.3. To Explore the Significant Lipid Metabolism Pathways and Gene by GSEA

**Figure 3:**
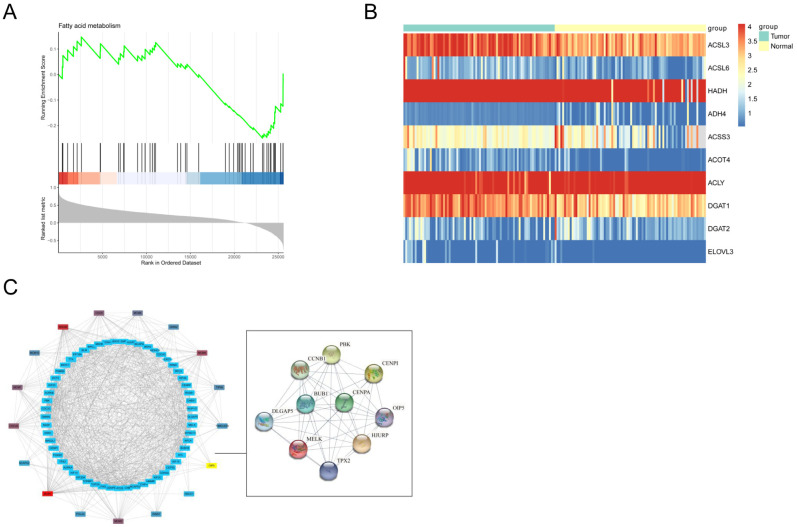
Identification of gene sets by GSEA. (**A**). The TCGA data used to map lipid metabolism-related heatmaps (**B**). Construction of a PPI network of lipid metabolism-related OIP5 genes (**C**).

Firstly, by calculating the correlation of the constructed PPI network, it was found that 20 highly centered genes, including OIP5, were key nodes, including CDC6, MCM6, GINS2, NDC80, MCM10, MCM7, CDC45, and MCM4. Next, in order to verify the role of lipid metabolism in the pathological process of ESCC, we analyzed the fatty acid-related pathway (fatty acid degradation) that was statistically significant using a GSEA assay. We found that only the OIP5 gene was significantly associated with the fatty acid degradation pathway among the 20 key nodes (NES value = −1.44406, *p* < 0.04) (Figure 3A). At the same time, we created a heatmap to visualize the difference in the expression of lipid metabolism-related genes between ESCC tissue and normal tissue. We found that ACSL3, ACSL6, HADH, and ADH4 enrichment was more pronounced in ESCC tissues than in normal tissues (Figure 3B). Eventually, we built the complex protein–protein interaction (PPI) network with OIP5 as a hub gene (Figure 3C). Through preliminary bioinformatics analysis, it was found that the hub gene OIP5 was significantly related to the fatty acid-metabolism pathway in the pathological process of ESCC. Therefore, we hypothesized that OIP5 may utilize fatty acids as an energy source for ESCC cell proliferation by influencing ACSLs. Furthermore, we hypothesized that OIP5 regulates fatty acid metabolism in ESCCs by affecting the expression of ACSLs, the key enzyme in lipid metabolism.

### 3.4. OIP5 Is Upregulated in ESCC and Correlated with Poor Prognosis in ESCC Patients

**Figure 4:**
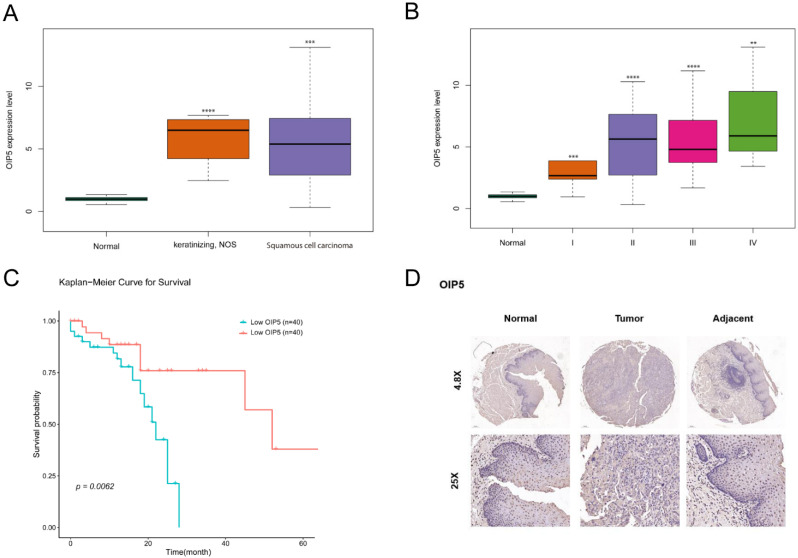
OIP5 was significantly upregulated in ESCC samples compared to adjacent non-cancer samples from TCGA database (*** *p* < 0.01, **** *p* < 0.001) (**A**). OIP5 mRNA expression level is increasing with different stages of ESCC tissues. (** *p* < 0.05, *** *p* < 0.01, **** *p* < 0.001) (**B**). Kaplan-Meier curves of OS differences according to the OIP5 mRNA expression level. (*p* = 0.0062) (**C**). The protein expression of OIP5 expression in tissue slides of ESCC through immunohistochemical staining (**D**).

By analyzing the TCGA dataset, we found that the expression of OIP5 was significantly upregulated in different ESCC tissue compared to that in normal tissues. The Kaplan-Meier survival analysis showed that the prognosis of the OIP5 high-expression group was worse than that of the OIP5 low-expression group, with a statistically significant difference. Furthermore, the expression pattern of OIP5 was found to gradually increase when the tumor pathological grade increased and then decreased (Stages II and III began to decline) (Figure 4B).

We determined the expression pattern and clinical significance of the central gene OIP5 in ESCC. We also found that patients with a high OIP5 expression had longer overall survival and better prognosis (Figure 4C), mainly because, firstly, false positives may occur in the differential and prognostic outcomes. Secondly, expression changed significantly in the early stages of cancer but not as much during cancer progression. Thirdly, a high expression of this gene was associated with the onset of cancer, and low expression was associated with cancer progression, suggesting that this gene does not play a role alone, but may be passively regulated.

### 3.5. OIP5 Expression Correlated with ESCC Patients Clinicopathological Features

The commercial ESCC-tissue chip used in this experiment includes 29 cases of ESCC tissue, the corresponding normal tissue around the carcinoma, and 2 cases of normal esophageal mucosal tissue for a total of 60 tissue cores. There were significant differences in the expression of OIP5 in ESCC tissue, adjacent tissue, and normal esophageal mucosa based on immunohistochemical staining results (χ2 = 29.03, *p* < 0.001)( Details are provided in Table 2). These were collected to further confirm the expression pattern of OIP5 with ESCC clinicopathological feature. The expression level of OIP5 was statistically significantly associated with tumor size (*p* = 0.026), T stage (*p* < 0.001), N stage (*p* < 0.011), and clinical stage (*p* < 0.001) in esophageal squamous cell carcinoma tissue, and no statistically significant difference was found between OIP5 expression and sex, age, M stage, and pathological grade (see Table 3). In other words, OIP5 gene is highly expressed in ESCC (Figure 4D).

### 3.6. OIP5 Increased the Expression Levels of Fatty Acid Metabolism-Related Enzymes and Intracellular Content in ESCC Cells

We first constructed lentiviral vectors for the downregulation and overexpression of the OIP5 and ACSL1 genes. ESC-410 and KYSE-150 ESCC cell lines were selected and divided into three groups: the control group (sh-NC), silenced-OIP5 gene group (sh-OIP5), and silenced-OIP5 group with an exogenous overexpression of OIP5 (sh-OIP5 + OIP5). The relative expression levels of OIP5 mRNA were examined separately. The same ESCC cell lines were also selected and divided into three groups: the control group (sh-NC), silenced-ACSL1 gene group (sh-ACSL1), and silenced-ACSL1 gene + overexpressed-ACSL1 gene group (Sh-ACSL1 + ACSL1). The relative expression levels of ACSL1 were also examined separately. We found that the mRNA expressions of the silenced-OIP5 gene group (sh-OIP5) and the silenced-ACSL1 gene group (sh-ACSL1) were significantly lower than those of the control group. On the contrary, when OIP5 and ACSL1 were overexpressed exogenously, their mRNA expression was significantly higher than that of the control group, indicating that our constructed lentiviral vector for the downregulation and overexpression of OIP5 and ACSL1 genes was successful and sufficient (Figure 4A).

**Figure 5:**
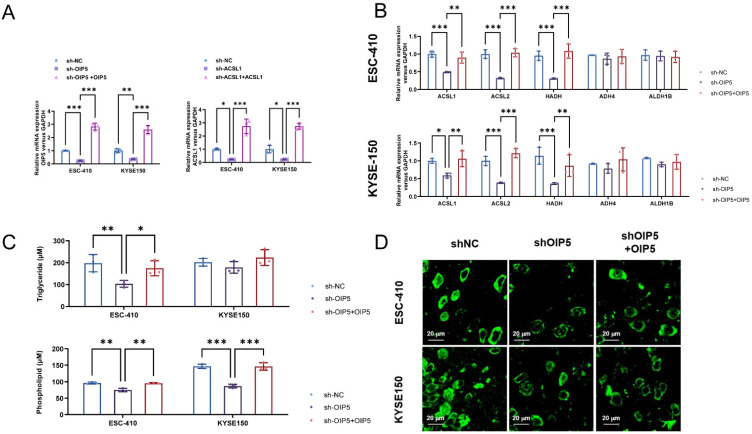
Construction of downregulation and overexpression of OIP5 and ACSL1 gene-carrying lentiviral vector. (* *p <* 0.05, ** *p <* 0.01, *** *p <* 0.001) (**A**). Changes in the mRNA expression levels of fatty acid-metabolism enzymes (ACSL1, ACSL2, HADH, ADH4, and ALDH1B) and (**B**) contents of triglycerides and phospholipids in (**C**) ESCC cells were detected by quantitative PCR by knocking down and overexpressing OIP5 in the ESC-410 and KYSE-150 cell lines. Intracellular neutral lipids in the ESC-410 and KYSE-150 cell lines detected using BODIPY 493/503 dye (**D**).

Firstly, ESC-410 and KYSE-150 ESCC cell lines were selected and divided into three groups: the control group (sh-NC), silenced-OIP5 gene group (sh-OIP5), and silenced-OIP5 group with exogenous overexpression of OIP5 (sh-OIP5 + OIP5). The mRNA expression levels of key lipid metabolism enzymes, ACSL1, ACSL2, HADH, ADH4, and ALDH1B, in the three groups were detected by Realtime PCR. Then, the content of related fatty acids and metabolic intermediates (such as phospholipids (PL) and triacylglycerol (TG)) in the three groups of lipid metabolic pathways were measured by mass spectrometry and fluorescent staining of neutral lipids. We found that silencing OIP5 gene group (sh-OIP5) significantly downregulated the mRNA expression levels of fatty acid metabolizing enzymes ACSL1, ACSL2, and HADH compared to the control group (sh-NC), while exogenous overexpression of OIP5 significantly restored these fatty acids’ expression of metabolic enzymes. In the ESC-410 and KYSE-150 cell lines, the knockdown and overexpression of OIP5 did not have a statistically significant influence on the mRNA levels of fatty acid metabolism enzymes (ADH4 and ALDH1B) compared to the control group (Figure 5B). Silencing of the OIP5-gene group (sh-OIP5) was found to significantly downregulate PLs and TGs levels compared to the control group (sh-NC) by quantitative PCR in ESC-410 cell lines, whereas restoration of OIP5 increased levels of TGs and PLs. For the KYSE-150 cell line, the sh-OIP5 group significantly downregulated the level of PLs compared with the sh-NC group, while the restoration of OIP5 increased the content of PLs. However, there was no statistically significant difference for TGs (Figure 5C). In addition, we also performed immunofluorescence staining on neutral lipids in the two cell lines, and we found that the content of neutral lipids in the sh-OIP5 group was significantly reduced, while overexpression of OIP5 restored the cellular neutral-lipid levels (Figure 5D). The results suggest that at the cellular level, OIP5 could promote the mRNA expression of fatty acid-metabolizing enzymes (ACSL1, ACSL2, and HADH), as well as the production of metabolic intermediates such as PL and TG, using neutral lipid fluorescence staining. In general, OIP5 plays an important role in the regulation of fatty acid metabolism in ESCC.

### 3.7. OIP5 Affects Lipid Metabolism in ESCC by Regulating the Fatty ACSL1 Enzyme

Fatty acids can be broken down into carbon dioxide and water in the body, and a large amount of energy can be released in the form of ATP and utilized by the organism. The chemical properties of exogenous or endogenous fatty acids are not active, and they need to be activated as fatty acyl-CoA outside the mitochondria of cells to enter the metabolic pathway. The long-chain acyl-coenzyme A synthetase family (ACSLs) is responsible for the activation of long-chain fatty acids, and it plays a major role in fatty acid metabolism. However, in cancer cells, its regulatory role is often deactivated, resulting in the change of the distribution and number of fatty acids, which can eventually lead to the occurrence of cancer and other metabolic diseases. Tumor cells may utilize fatty acids as an energy source for cell proliferation by expressing ACSLs. Preliminary bioinformatics analysis suggests that OIP5 is involved in the regulation of fatty acid metabolism in ESCC, but the detailed mechanism remains unclear. In preliminary experiments, we found that the expression of ACSL1, the key enzyme of fatty acid activation in the first step of lipid metabolism in ESCC, was significantly affected by the expression of OIP5. We further hypothesized that OIP5 regulates fatty acid metabolism in ESCC by affecting the expression of ACSL1, a key enzyme in lipid metabolism.

**Figure 6:**
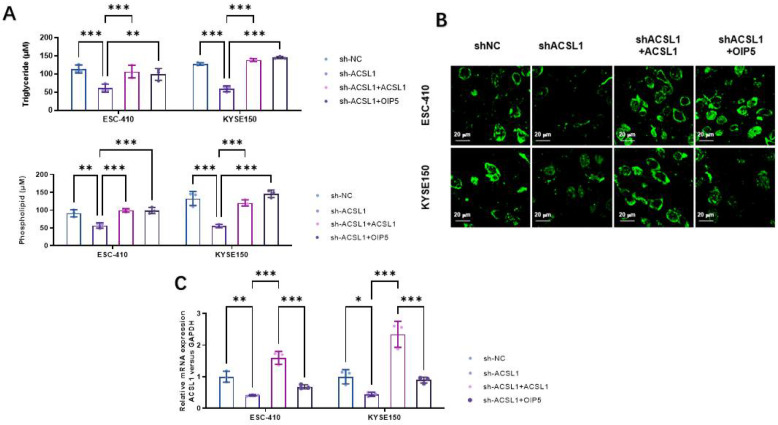
The contents of triglycerides and phospholipids in ESCC cells were detected by quantitative PCR by knocking down and overexpressing OIP5 in the ESC-410 and KYSE-150 cell lines (**A**). Intracellular neutral lipids detected using BODIPY 493/503 dye in the ESC-410 and KYSE-150 cell lines (**B**). RT-qPCR analysis for ACSL1 mRNA levels in the ESC-410 and KYSE150 cell lines (**C**). (* *p <* 0.05, ** *p <* 0.01, *** *p <* 0.001).

Secondly, to further demonstrate the relationship between ACSL1 and OIP5, we selected the same ESCC cell lines and divided them into four groups including control group (sh-NC), silenced-ACSL1 gene group (sh-ACSL1), silenced-ACSL1 gene + overexpressing-ACSL1 genome (Sh-ACSL1 + ACSL1), and silenced-ACSL1 gene + OIP5-overexpression group (Sh-ACSL1 + OIP5). By utilizing mass spectrometry analysis of metabolic intermediates in lipid metabolism-pathway (such as PLs and TGs) content assay and fluorescent staining of neutral lipids, we found that in the ESC-410 and KYSE-150 cell lines, the TGs and PLs content of the sh-ACSL1 group were significantly reduced compared with the control group, while in the silenced-ACSL1 gene + exogenous overexpressing-ACSL1 genome (Sh-ACSL1 + ACSL1), silencing the ACSL1 gene + exogenous overexpressing-OIP5 gene (Sh-ACSL1 + OIP5) restored TGs and PLs in ESCC cell lines to a certain level (Figure 6A,B). We also examined the mRNA expression levels of ACSL1 in the four groups of tumor tissues by Realtime PCR. In the ESC-410 and KYSE-150 cell lines with a silenced-ACSL1 genome (sh-ACSL1), the exogenous overexpression of OIP5 can restore ACSL1-mRNA expression, demonstrating that OIP5 promoted lipid metabolism at the cellular expression level of the key enzyme ACSL1 to further regulate fatty acid metabolism in ESCC (Figure 6C).

### 3.8. OIP5 Promotes ESCC Development and Regulate Fatty Acid Metabolism by ACSL1 In Vivo and In Vitro

**Figure 7:**
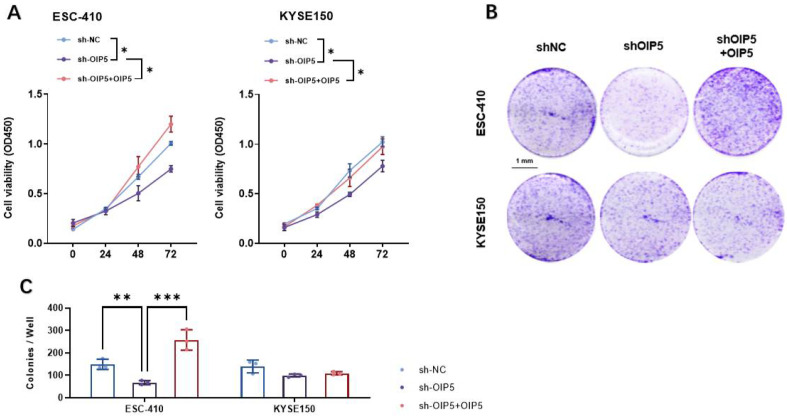
The proliferation level of ESC-410 and KYSE-150 cells was detected and analyzed by CCK-8 methods (**A**). The colony-forming ability of ESC-410 and KYSE-150 cells was detected by colony formation assay (**B**), and the corresponding bar graphs were made (**C**). (* *p <* 0.05, ** *p <* 0.01, *** *p <* 0.001).

To evaluate the tumorigenic ability of OIP5 in ESCC, the same ESCC cell lines described above were selected and divided into three groups including, the control group (sh-NC), silenced-OIP5 gene group (sh-OIP5), and silenced-OIP5 gene group with an exogenous overexpression of OIP5 gene (sh-OIP5 + OIP5). The proliferation levels of the three groups of cell lines at 0, 24, 48, and 72 h were detected by CCK8 (Figure 7A), and the clone-formation ability of the cells was detected by the clone-formation assay. The silenced-OIP5 gene group (sh-OIP5) in the ESC-410 cell line significantly reduced cell viability and significantly decreased the number of clones compared to the control group (sh-NC). On the other hand, the exogenous overexpression of the OIP5 gene after silencing the OIP5 gene group (sh-OIP5 + OIP5) significantly reversed this phenomenon (Figure 7B,C). Cell viability was significantly higher, and the number of clones was significantly increased in the sh-OIP5 + OIP5 group compared to the control group. In KYSE150, cell viability was significantly downregulated, and the clonogenic number was significantly reduced in sh-OIP5 compared to the control group, but the changes in cell viability and clonogenic number were not significant in the sh-OIP5 + OIP5 group compared to the control group. Restoration of OIP5 expression partially upregulated cell viability and promoted colony formation in ESCC (Figure 7B,C). Therefore, the above results suggest that the upregulation of OIP5 expression is a tumor-promoting factor in esophageal squamous cell carcinoma cells.

Thirdly, we investigated in vitro and at the animal level whether OIP5 regulates fatty acid metabolism by affecting the expression of ACSL1.

**Figure 8:**
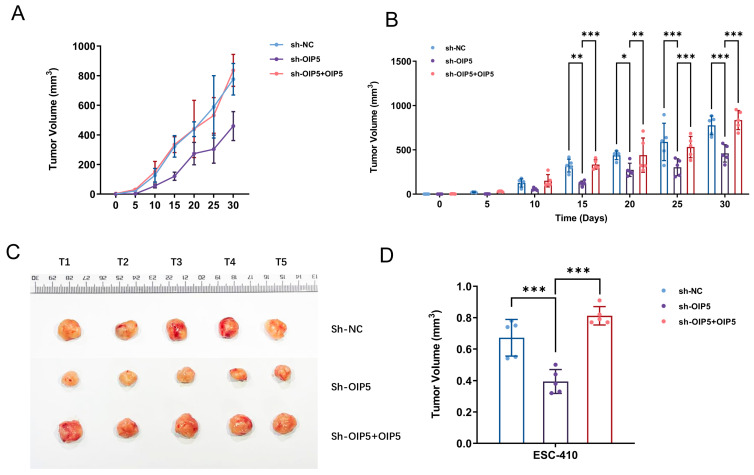

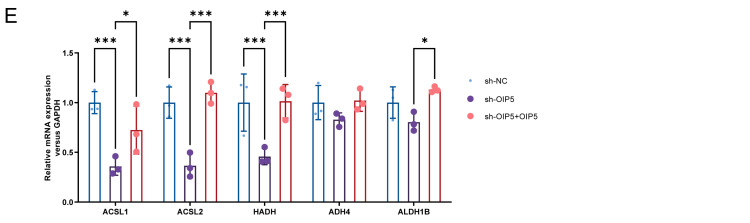
Subcutaneous tumor growth curves of mice in sh-NC, sh-OIP5, and sh-OIP5 + OIP5 experimental groups (**A**). The data in panel A are recreated as histograms of subcutaneous tumor volume growth in mice in the experimental groups to facilitate the visualization of statistical significance (**B**). Representative pictures of dissected tumors of nude mice transplanted with ESC-410 ESCC cells (**C**). Histogram of subcutaneous tumor-volume growth of mice in different experimental groups (**D**). Relative mRNA-expression patterns of OIP5 under lipid-metabolism genes expressed differently in nude-mouse tumor tissues (**E**). (* *p <* 0.05, ** *p <* 0.01, *** *p <* 0.001).

We constructed a tumorigenic model in nude mice and selected the ESC-410 cell line for subcutaneous injection into nude mice. We found that the silenced-OIP5 gene group (sh-OIP5) grew slower, smaller, and had a lighter tumor weight compared to normal controls, and the exogenous restoration of OIP5 expression significantly reversed these phenomena (Figure 8A–D). The results suggest that OIP5 can promote the occurrence and development of ESCC in vitro. We further investigated the relative mRNA expression patterns of lipid metabolism-related genes in nude-mouse tumor tissues. It was found that OIP5 silencing in nude-mouse tumor tissues not only slowed tumor growth and reduced size, but also significantly downregulated the expression of fatty acid pathway-related genes (ACSL1, ACSL2, and HADH). The heatmap in the previous bioinformation analysis also illustrated this phenomenon (Figure 3B). However, restoring the expression of OIP5 reversed the expression levels of the above genes, not only promoting tumor growth, but also upregulating the expression pattern of fatty acid-metabolism enzymes. The above experimental phenomena demonstrates that the upregulation of OIP5 gene can promote the expression of lipid metabolism-related enzymes and ESCC development in vitro and in vivo (Figure 8E).

## 4. Discussion

Despite improvements in surgical techniques and adjuvant chemoradiotherapy, esophageal squamous cell carcinoma has a poor prognosis among malignancies. The pathogenesis of esophageal cancer has not been thoroughly investigated, but abnormal gene expression is still considered as an important factor in the occurrence of esophageal cancer. Gene expression profiling (also known as transcriptomics) enables the detection of entire genomes, which may help elucidate tumorigenesis. Metabolomics provides a wealth of data information reflecting genetic and epigenetic metabolic alterations and is dedicated to the identification and development of metabolically active targets in cancer therapeutics and pharmacology [12]. Thus, the integration of metabolomics with transcriptomics may provide a deeper understanding of tumor pathogenesis [13].

OIP5 is a protein-coding gene located on chromosome 15 and belongs to the cancer/testis antigen (CTA) gene family [7]. OIP5 encodes a 25 kDa protein with a helical-structured domain, originally identified as an Opa (Neisseria gonorrhoeae opacity-associated) interacting protein by a yeast two-hybrid analysis [14]. Aberrant OIP5 expression is common in various types of cancer, including glioblastoma [15], bladder, esophagus [16], breast [17], gastric, colorectal [10], and liver cancer [18]. The expression status and biological function of OIP5 in esophageal squamous cell carcinoma and the exact mechanism of OIP5 have not been well studied.

In the early stage, we obtained differentially expressed genes (DEGs) in esophageal squamous cell carcinoma by analyzing the GEO and TCGA databases, specifically, we analyzed the TCGA dataset by gene ensemble enrichment analysis (GSEA). We found that OIP5 in differentially expressed genes was significantly associated with fatty acid-metabolism pathways. OIP5 was identified as the central gene, and the PPI network was constructed. Epidemiological studies have shown that the risk of esophageal cancer in obese people is increased, and previous literature has reported that fatty acid metabolism is closely related to the occurrence and development of malignant tumors. This suggests that the central gene OIP5 may affect the fatty acid metabolism of esophageal squamous cell carcinoma and the occurrence and development of esophageal squamous cell carcinoma. We found that the expression of ASCL1, a key enzyme for the activation of fatty acids in ESCC, correlated significantly with the expression pattern of OIP5 through preliminary experiments. OIP5 silencing or overexpression alters the content of fatty acids and their metabolic intermediates such as PLs and TGs in ESCC cells by affecting the expression of ACSL1. Through in vitro cell experiments and tumorigenic experiments in nude mice, it was elucidated that OIP5 affects fatty acid metabolism in mice by promoting the expression of ACSL1. OIP5 is a tumor-promoting factor of esophageal squamous cell carcinoma cells, which can promote the occurrence and development of esophageal squamous cell carcinoma. Our research is of great significance for elucidating the occurrence of ESCC by identifying the central genes and key pathways of esophageal cancer and may provide new ideas for the diagnosis and treatment of ESCC. It is expected to improve the ESCC prognostic model, elucidate the drug-resistance mechanism, and develop corresponding targets to provide a rationale for treatment.

This study also has certain limitations and shortcomings, such as those of the results we obtained from the knockdown and exogenous overexpression in two ESCC cell lines of several common fatty acid-metabolizing enzymes ACSL1, ACSL2, HADH, ADH4, and ALDH1B. OIP5 did not significantly downregulate and restore the mRNA expression of ADH4 and ALDH1B. We speculate that this may be due to experimental error or because ADH4 and ALDH1B are not the key fatty acid-metabolizing enzymes for OIP5-mediated lipid metabolism in ESCC. When triglycerides, phospholipids, and neutral lipids were used as the detection targets, satisfactory positive results were obtained regardless of OIP5 knockdown or overexpression. Triglyceride, phospholipid, and neutral lipid content were significantly recovered. This indicates that OIP5 is inseparable from ESCC lipid metabolism. Although our design is well-established, there are still some inconsistencies in the experimental results. In addition, the number of ESCC tissue chips we purchased was small, and the difference between immunohistochemical ESCC and normal tissues made by the tissue chips was not significant. The conclusion that OIP5 is related to the clinicopathological factors of ESCC needs to be further confirmed by larger samples. This study can provide a basis for further verification and research for new ESCC central genes for later generations.

## 5. Conclusions

In conclusion, our study demonstrated for the first time that OIP5 is a central gene and an important oncogenic factor in ESCC that is closely related to fatty acid metabolism. On one hand, we determined the expression pattern and clinical value of the central gene OIP5 in ESCC by comparing the differential expression levels of OIP5 in ESCC tissues and adjacent control tissues and provided correlating statistical analysis of case data. On the other hand, the effects of OIP5 on the fatty acid metabolism and tumorigenicity of ESCC were analyzed by constructing lentiviral vectors for the downregulation and overexpression of the OIP5 and ACSL1 genes. Last but not least, the role of OIP5 in the lipid metabolism of ESCC was elucidated by in vitro cellular assays and tumorigenicity assays in nude mice.

## Figures and Tables

**Table 1:** Primer sequences were designed using Primer Premier, as shown in the table below.

Gene	Forward (5′-3′)	Reverse (5′-3′)
OIP5	GGGGGACTTTTGTGGTGGCA	ACTGGAACACAGCGCACCTC
ACSL1	AACAACAGCCTGTGGGACCG	CAAATTGCACGGCATCGGGG
ACSL2	AACCGCGTGAAGCTGGTGAA	GCGGATGTACTCCACGGCTG
HADH	ATAGCGACCAGCACGGATGC	AATGGAGGCCAGCGAATCGG
ADH4	GCCAAGGTCACCCCTGGTTC	CACCTGCACAGTCAAGGGCA
ALDH1B	GTGGCCAGATCATCCCGTGG	GGTGGAACCGGTGAAGGCAA
GAPDH	GCGGGGCTCTCCAGAACATC	TCCACCACTGACACGTTGGC

**Table 2:** OIP5 expressed in tissue chips of ESCC through IHC.

		OIP5				*p*
		−	+	++	+++	
Cases	Normal	1	26	1	1	<0.0001
	Tumor	0	3	11	15	

−, +, ++, and +++ represents different levels of the OIP5 protein expression level, which was negative, slight-positive, moderate-positive and strong-positive in tissue chips.

**Table 3:** The relationship between the OIP5 expression and different clinicopathological characteristic of ESCC (Number of tissue chips = 29).

	Overall	+	++	+++	*p*
*n*	29	3	11	15	
Gender = 0/1 (%)	9/20 (31.0/69.0)	0/3 (0.0/100.0)	3/8 (27.3/72.7)	6/9 (40.0/60.0)	0.37
Age (mean (SD))	62.79 (7.86)	70.33 (11.06)	64.09 (6.55)	60.33 (7.44)	0.101
Tumor size (mean (SD))	3.86 (1.10)	2.60 (1.68)	3.61 (1.03)	4.30 (0.82)	0.026
T stage (%)					<0.001
1	2 (7.4)	2 (66.7)	0 (0.0)	0 (0.0)	
2	8 (29.6)	1 (33.3)	6 (54.5)	1 (7.7)	
3	16 (59.3)	0 (0.0)	5 (45.5)	11 (84.6)	
4	1 (3.7)	0 (0.0)	0 (0.0)	1 (7.7)	
N stage (%)					0.011
0	12 (41.4)	3 (100.0)	8 (72.7)	1 (6.7)	
1	9 (31.0)	0 (0.0)	2 (18.2)	7 (46.7)	
2	5 (17.2)	0 (0.0)	1 (9.1)	4 (26.7)	
3	3 (10.3)	0 (0.0)	0 (0.0)	3 (20.0)	
M stage = 0/1 (%)	27/2 (93.1/6.9)	3/0 (100.0/0.0)	11/0 (100.0/0.0)	13/2 (86.7/13.3)	0.594
Pathological grade (%)					0.574
I	5 (17.2)	0 (0.0)	2 (18.2)	3 (20.0)	
II	22 (75.9)	3 (100.0)	9 (81.8)	10 (66.7)	
III	2 (6.9)	0 (0.0)	0 (0.0)	2 (13.3)	
Clinical stage (%)					<0.001
1	2 (6.9)	2 (66.7)	0 (0.0)	0 (0.0)	
2	12 (41.4)	1 (33.3)	9 (81.8)	2 (13.3)	
3	13 (44.8)	0 (0.0)	2 (18.2)	11 (73.3)	
4	2 (6.9)	0 (0.0)	0 (0.0)	2 (13.3)	
Lymph number (median [IQR])	7.50 [4.75, 11.00]	9.00 [8.00, 10.50]	9.00 [6.25, 11.00]	6.00 [3.50, 10.50]	0.455

## Data Availability

The raw data supporting the conclusions of this article will be made available by the authors, without undue reservation.

## Abbreviations

ESCC	esophageal squamous cell carcinoma
DEGs	differentially expressed genes
GSEA	gene set enrichment analysis
TCGA	The Cancer Genome Atlas
ACSL1	acyl-CoA synthetase long-chain family member 1
EAC	esophageal adenocarcinoma
OIP5	Opa-interacting protein 5
GO	Gene Ontology
KEGG	Kyoto Gene and Genomic Encyclopedia
PPI	The protein-protein interaction
CCLE	Cancer Cell Line Encyclopedia
STRING	Search Tool for the Retrieval of Interacting Genes
CTA	cancer/testis antigen
